# Do Body Mass Index and Nutritional Risk Score 2002 Influence the In-Hospital Mortality of Patients Following Cardiac Arrest?

**DOI:** 10.3390/nu15020436

**Published:** 2023-01-14

**Authors:** Piotr Fehler, Marzena Zielińska, Bartosz Uchmanowicz, Raúl Juárez-Vela, Łukasz Lewandowski, Stanisław Zieliński, Michał Czapla

**Affiliations:** 1 Institute of Heart Diseases, University Hospital, 50-566 Wrocław, Poland; 2 Department of Anaesthesiology and Intensive Care, University Hospital, 50-556 Wrocław, Poland; 3 Department and Clinic of Anaesthesiology and Intensive Therapy, Faculty of Medicine, Wrocław Medical University, 50-556 Wrocław, Poland; 4 Department of Nursing and Obstetrics, Faculty of Health Sciences, Wrocław Medical University, 51-618 Wrocław, Poland; 5 Group of Research in Care (GRUPAC), Faculty of Health Sciences, University of La Rioja, 26006 Logroño, Spain; 6 Department of Medical Biochemistry, Wrocław Medical University, 50-368 Wrocław, Poland; 7 Department of Emergency Medical Service, Wrocław Medical University, 51-616 Wrocław, Poland

**Keywords:** obesity, sudden cardiac arrest, mortality, BMI, ICU, ROSC

## Abstract

Background: Contemporarily, cardiac arrest (CA) remains one of the leading causes of death. Poor nutritional status can increase the post-CA mortality risk. The aim of this study was to determine the relationship between body mass index (BMI) and Nutritional Risk Score 2002 (NRS 2002) results and in-hospital mortality in patients admitted to the intensive care unit (ICU) after in-hospital and out-of-hospital cardiac arrest. Methods: A retrospective study and analysis of medical records of 161 patients admitted to the ICU of the University Clinical Hospital in Wrocław (Wrocław, Poland) was conducted. Results: No significant differences in body mass index (BMI) and nutritional risk score (NRS 2002) values were observed between non-survivors and survivors. Non-survivors had significantly lower albumin concentration (*p* = 0.017) and total cholesterol (TC) (*p* = 0.015). In multivariate analysis BMI and NRS 2002 scores were not, per se, associated with the in-hospital mortality defined as the odds of death (Model 1: *p*: 0.700, 0.430; Model 2: *p*: 0.576, 0.599). Univariate analysis revealed significant associations between the hazard ratio (HR) and TG (*p* ≈ 0.017, HR: 0.23) and hsCRP (*p* ≈ 0.018, HR: 0.34). In multivariate analysis, mortality risk over time was influenced by higher scores in parameters such as BMI (HR = 0.164; *p* = 0.048) and hsCRP (HR = 1.006, *p* = 0.002). Conclusions: BMI and NRS 2002, on their own (unconditionally – in the whole study group) did not alter the odds of mortality in patients admitted to the intensive care unit (ICU) after in-hospital and out-of-hospital cardiac arrest. The risk of in-hospital mortality (expressed as hazard ratio – the risk over the time period of the study) increased with an increase in BMI but not with NRS 2002.

## 1. Introduction

Cardiac arrest (CA) is one of the leading causes of death in the developed world [1]. Poor nutritional status can increase mortality risk following CA events. Furthermore, overweight and obesity are known to be associated with poor neurological outcomes in patients following CA [2]. Overweight and obesity pose a problem faced by 53 percent of the European Union’s population [3]. Undoubtedly, the lack of proper BMI maintenance is one of the more serious global public health problems [4]. Obesity is often associated with various comorbidities that can not only directly threaten the health and/or life of patients but also determine their prognosis [5,6]. The fact that overweight and obesity are a cause of diabetes mellitus (DM), hypertension (HT), hyperlipemia, cardiovascular disease (CVD), and certain cancers is well-documented in the form of numerous study reports [7,8,9]. When it comes to the COVID-19 pandemic, obesity has also been identified as one of the factors promoting the severe course of the disease [10,11]. Patients suffering from this condition may experience complications related to more frequent respiratory distress resulting from reduced chest and lung compliance and respiratory muscle failure, interalia [12]. Moreover, abnormal body mass can also pose a problem during patient intubation and extubation. Obese patients show a greater tendency towards developing the collapse of the upper airway; thus, they are more likely to require reintubation. The hospitalization of obese patients is prone to prolongation due to the aforementioned fact [13]. In Europe, the algorithm for medical specialists to manage CA is defined by the European Resuscitation Council Guidelines. When the initiating rhythm of CA is asystole/electrical activity without pulse (PEA), the patient requires both cardiopulmonary resuscitation and drug administration (e.g., epinephrine). When the initiating rhythm is ventricular fibrillation (VF)/pulseless ventricular tachycardia (pVT), the patient requires additional defibrillation [14]. Interestingly, performing subsequent chest compressions and defibrillation acts during the CA may be less effective due to the presence of fatty tissue on the anterior and posterior subcutaneous adipose tissues [15,16]. Despite the presence of a vast number of disorders, many researchers describe a so-called “obesity paradox” [17], linked to better outcomes in the out-of-hospital cardiac arrest (OHCA) cohort, as well as improved survivability among patients suffering from heart diseases such as acute coronary syndrome (ACS) and heart failure (HF) [18,19]. Malnutrition acts as another factor that is associated with higher risk in hospital mortality and longer hospitalization in the intensive care unit (ICU) [20]. However, studies linking malnutrition to perilous ICU clinical outcomes have shown discrepant results, partly due to inadequate diagnoses of malnutrition [21]. According to current legislation in Poland, every patient must undergo a nutritional assessment upon hospital admission, with the use of screening tools such as the Nutritional Risk Score 2002 (NRS 2002) or the Subjective Global Assessment, being in line with the Global Leadership Initiative on Malnutrition guidelines [22].

The aim of this study was to determine the relationship between some components of nutritional status and in-hospital mortality in patients admitted to the ICU after in-hospital and out-of-hospital cardiac arrest. This question was addressed through the following specific objectives of the study:

- To assess whether there is a relationship between survival and BMI score;
- To assess whether there is an association between survival, malnutrition risk, and high malnutrition risk according to the NRS 2002.

## 2. Materials and Methods

### 2.1. Study Design and Setting

The medical records of 161 patients admitted for SCA (ICD10: I46) to the ICU of the University Clinical Hospital in Wrocław between January 2017 and February 2022 were analyzed retrospectively.

### 2.2. Study Population and Data

All patients included in the study met the maturity criterion (age: 18 or older) and were admitted to the ICU because of CA that did not result from excessive trauma. Patient data on length of hospitalization, BMI, NRS 2002 score, comorbidities, and laboratory results were collected. The study group was divided in two ways. The first division of patients was into one of three groups according to WHO classification: normal weight (BMI 18.5–24.9), pre-obese (BMI 25–29.9), and obese (BMI ≥ 30). There were no individuals in the study group with a BMI of < 18.5. An alternative form of grouping, used as an auxiliary assessment of differences in the values of continuous variables, was based on the following cut-off BMI values: non-obese (BMI < 30) and obese (BMI ≥ 30). The patients were grouped with respect to nutritional status against the NRS 2002 cut-off value of 5. Values ≥ 3 indicated the risk of malnutrition. Conversely, values ≥ 5 indicated high risk of malnutrition [23]. The BMI and NRS 2002 scores of each individual patient were procured and updated by the physician who had admitted the patient to the ward.

### 2.3. Ethical Considerations

The study was conducted following the principles of the Declaration of Helsinki and was approved by the independent Bioethics Committee of Wrocław Medical University (No. KB-776/2022). The study followed the STROBE guidelines (Strengthening the Reporting of Observational Studies in Epidemiology).

### 2.4. Statistical Analysis

Data pre-processing and visualization were performed with Python 3.9.13. Statistical analysis was performed with Python 3.9.13 or the STATISTICA 13.3 package on license by Wrocław Medical University. The following Python packages were utilized: numpy 1.23.0, pandas 1.4.3, scikit-learn 1.1.3, scipy 1.9.3, statsmodels 0.13.2, zepid 0.9.1, and matplotlib 3.6.0. Statistical inference was based on α = 0.05.

Analysis of the distribution of values of the selected variables, their scale of measurement, and the incidence of outliers or extreme values in the dataset were taken into account when selecting the most suitable methods for statistical inference. Differences between values after grouping by different categories were checked with the use of Kruskal–Wallis ANOVA or the Mann–Whitney U test, depending on the count of categories.

The contingency tables were analyzed with the Χ2 test. In the case of the 2 × 3 tables, if the *p*-value from this test was lower than the α value, subsequent pairwise Χ2 tests were performed after decomposing the tables into 2 × 2 tables. The *p*-values obtained from these pairwise tests were corrected for a false discovery rate of 0.05 with the Benjamini–Hochberg method and were used in a post-hoc analysis.

The incidence of death among the population sample was modeled by logistic regression with sigma-restricted (quasi-experimental) coding. Only main effects were used in the multivariate models. Stepwise elimination (*p* cutoff = 0.10) was used to obtain the multivariate model with the most impactful main effects. Apart from BMI and albumin, which were used in the analysis, variables with more than 5% of the data missing were excluded from the base variables used in the iterative selection of the most significant effects in order to prevent a loss of input data to the model and its associated excessive bias. Two custom multivariate models were used to assess the association between nutritional status and the odds of death. The assumption of linearity between the predictors and the logit was checked with the Box–Tidwell test. Goodness of fit was assessed with the Hosmer–Lemeshow test, the Akaike information criterion (AIC), the Bayesian information criterion, and Nagelkerke’s pseudo-R2. The hypothesis that β = 0 was tested with the Lagrange multiplier (score) test. Prediction power was analyzed based on the assessment of testing AUC computed with tenfold cross-validation.

Survival analysis was performed using the Cox proportional hazards regression model based on the Breslow estimator, with σ-restricted parametrization. The proportional hazards assumption was evaluated with the Schoenfeld test.

## 3. Results

### 3.1. Characteristics of the Sample Population

In order to analyze differences in the values of the continuous variables, the sample population was divided into groups. After stratifying the patients by BMI value, no significant differences (apart from those in BMI values) were found (Table 1).

In case of division in context or undernutrition state according to NRS 2002, patients with NRS ≥ 5 showed lower median BMI values compared to patients of lower risk of undernutrition (*p* ≈ 0.040) (Table 2). Interestingly, differences between non-obese (BMI < 30) and obese patients were observed not only in BMI (*p* < 0.001), but also age (*p* ≈ 0.039) and plasma potassium concentration (*p* ≈ 0.021) (Table 2).

The numbers of patients with different BMI values, after accounting for comorbidities, are shown in Table 3. Significant differences in frequency were observed for chronic kidney disease (CKD) (*p* ≈ 0.034), diabetes mellitus (DM) (*p* < 0.001), and hypertension (HT) (*p* ≈ 0.017). Obese patients were characterized by 4.7-fold (*p* ≈ 0.031) higher odds of CKD compared to patients with a BMI of < 30. Patients with proper BMI score, in comparison with patients with a BMI of ≥25, showed 3.62-fold (*p* ≈ 0.017) lower odds of DM and 2.97-fold (*p* ≈ 0.020) lower odds of HT.

### 3.2. Survival Analysis

#### 3.2.1. Differences in Selected Parameters and Comorbidity in the Context of Survival

**Figure 1:**
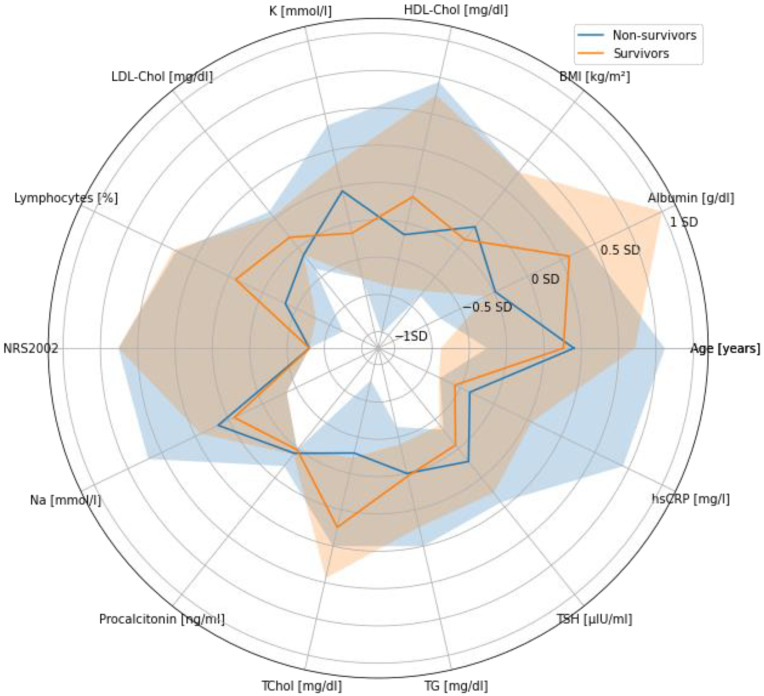
Radar plot of standardized values of selected quantitative parameters in the context of survival. Median values and 1st-to-3rd quartile range are marked with lines and colored areas, respectively. Abbreviations: BMI, body mass index; HDL, high-density lipoprotein; K, potassium; LDL, low-density lipoprotein; NRS 2002, Nutritional Risk score; Na, sodium; PCT, procalcitonin; TC, total cholesterol; TG, triglycerides; TSH, thyroid-stimulating hormone; hsCRP, high-sensitivity *C-*reactive protein.

No significant differences in BMI values were observed between non-survivors and survivors (*p* ≈ 0.632). However, these strata showed different serum albumin concentrations (*p* ≈ 0.017) (Table 4). Death occurred more frequently among patients with lower albumin concentration (Figure 1). Likewise, serum concentrations of TC (*p* ≈ 0.015) and PCT (*p* ≈ 0.006) varied significantly between non-survivors and survivors. Patients with higher TC concentration survived more frequently. Interestingly, the median value non-survivor-to-survivor ratio of PCT concentration was 4:1. The descriptive statistics of all analyzed parameters are shown in Table 4 and Figure 1.

**Figure 2:**
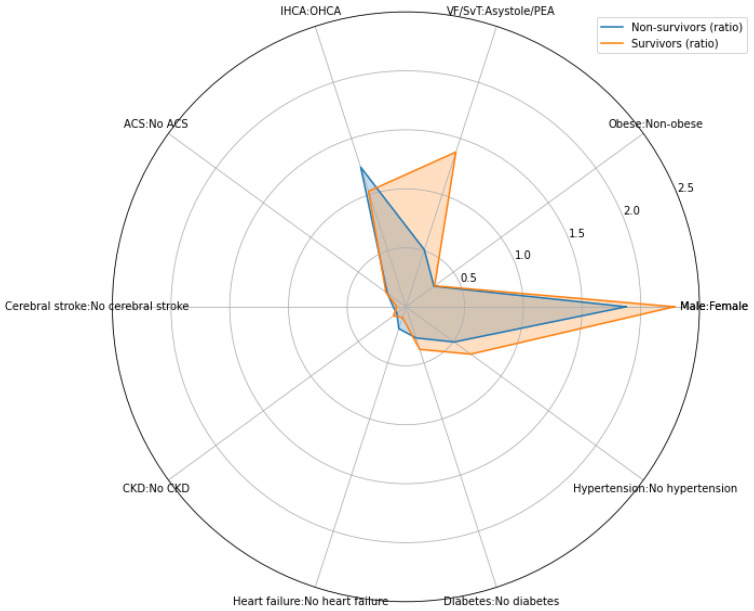
Radar plot of comorbidity incidence and sex. Lines and colored areas mark the count ratios. ACS, acute coronary syndrome; HF, heart failure; DM, diabetes mellitus; CKD, chronic kidney disease; CS, cerebral stroke; HT, hypertension; OHCA, out-of-hospital cardiac arrest; IHCA, in-hospital cardiac arrest; PEA, pulseless electrical activity; VF, ventricular fibrillation (VF); pVT, pulseless ventricular tachycardia.

The only statistically significant difference regarding the comorbidities was associated with the cardiac arrest mechanism (*p* ≈ 0.002). The odds ratio of death for patients with asystole/PEA to patients with VF/pVT was 2.71 (Table 5, Figure 2).

#### 3.2.2. Modeling Mortality Incidence with Logistic Regression

**Figure 3:**
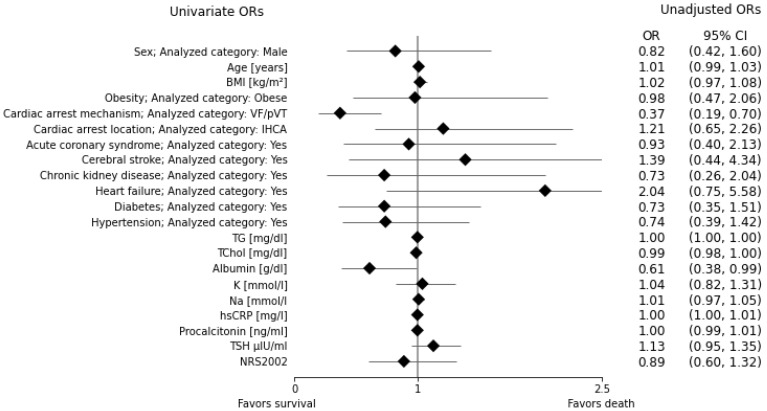
Forest plot of univariate odds ratios (ORs).

The univariate data analysis showed 2.72-fold higher odds (*p* ≈ 0.0021) of death among patients with the asystole/PEA cardiac arrest mechanism compared to patients with the VF/pVT mechanism. Interestingly, the odds of death decreased 1.63-fold (*p* ≈ 0.048) for each g/dL increase in serum albumin concentration. The univariate odds ratios for the analyzed variables are shown in Figure 3 and Table S1 (Supplementary Materials).

Multivariate analysis provided more information on the dependence of death on selected factors (Table 6). The custom multivariate models featured parameters associated with nutritional status, either exclusively (Table 6: Model 1) or including sex and age (Table 6: Model 2). In both models, BMI and NRS 2002 scores were insignificant in the context of survival status (*p*: 0.700, 0.430 and *p*: 0.576, 0.599 for Models 1 and 2, respectively). The first model (Table 6: Model 1) had severely suboptimal prediction accuracy, correctly predicting approximately 41.3% of survival statuses in the test dataset. Moreover, the addition of sex and age information to this model (Table 6: Model 2) impaired the prediction accuracy (approximately 37.9% accuracy).

The model selected through iteration (Table 6: Model 3) utilized three effects: cardiac arrest mechanism (*p* ≈ 0.038), hsCRP (*p* ≈ 0.038), and incidence of heart failure (*p* ≈ 0.069). Patients with the VF/pVT cardiac arrest type had 1.68-fold lower odds of death than patients with the asystole/PEA cardiac arrest type. Moreover, with higher hsCRP serum concentration, the odds of death increased by 0.9% for every unit (1 g/dL). The model had moderate prediction accuracy, correctly predicting survival status in approximately 66% of patients. The iterative selection of main effects used in this model is shown in the Supplementary Materials (Table S2).

Based on pseudo-R^2^ values, the model that uses the information on hsCRP, cardiac arrest mechanism, and incidence of heart failure (Table 6: Model 3) indisputably had the best ability to predict death in the dataset (R^2^ ≈ 0.2042 in Model 3 vs. R^2^ = 0.0085 and 0.0236 in Models 1 and 2, respectively).

#### 3.2.3. Survival Analysis with Cox Proportional Hazards Regression

Univariate analysis (Supplementary Materials: Table S3) revealed significant associations between the hazard ratio (HR) and serum concentrations of TG (*p* ≈ 0.017) or hsCRP (*p* ≈ 0.018). An increase in these parameters by 1 mg/dL (TG) or 1 mg/l (hsCRP) was associated with an increase in HR by 0.23% or 0.34%, respectively.

**Figure 4:**
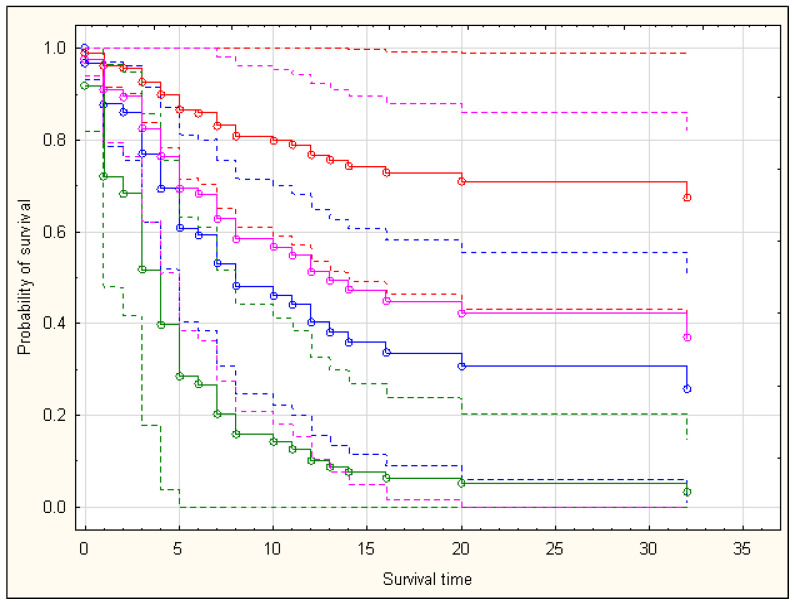
Survival curves for people with a serum hsCRP concentration of 49.02 mg/L (mean value among the sample) and different BMI values and incidence of diabetes. The colors blue, red, green, and pink indicate the following patient parameters, respectively: BMI = 20 and no diabetes, BMI = 20 and diabetes, BMI = 35 and no diabetes, and BMI = 35 and diabetes. Values of other features used by the model (Table 7) were set as equal for all of the four curves in order to visualize the differences in survival curves associated with variable BMI and diabetes comorbidity status. Probability of survival was determined with use of the Breslow estimator.

According to the multivariate model shown in Table 7, a one-unit increase in BMI or hsCRP (in mg/L) was associated with an increase in HR by 6.37% (*p* ≈ 0.048) or 0.60% (*p* ≈ 0.002), respectively. Moreover, the incidence of diabetes decreased the values of the hazard function 3.44-fold. Exemplary survival curves are shown in Figure 4.

## 4. Discussion

In this study, both BMI and NRS 2002 results had no clear impact on the survival of patients admitted to the ICU after in-hospital and out-of-hospital cardiac arrest. The evidence regarding the relationship between BMI score and mortality is conflicting. Some authors show no significant association with BMI, while others report an increase or decrease in patient mortality [24]. In this study, there was no significant difference in BMI values between survivors and non-survivors. Likewise, BMI could not, on its own, be utilized as factor used in estimation of the odds of death during ICU stay. However, the risk in hospital mortality increased by 6.37% for each unit increase in BMI over time. The issue of the impact of obesity on the length of hospital stay and in-hospital mortality within the ICU is controversial [25]. Matinrazmt et al. found that obesity was associated with lower mortality risk in a similar group of patients (HR: 0.86 increase per 1 BMI category) [26]. The “obesity paradox” is a well-known phenomenon among patients with heart failure or acute coronary syndrome (among others), although the mechanisms of this paradox remain speculative [11,19]. In addition, Pepper et al., in their meta-analysis, pointed out that patients admitted to the ICU with sepsis with coexistent overweight or obesity (identified with BMI values) expressed reduced adjusted mortality [27].

Authors often rely on the BMI score, although the indicator itself is flawed. One of the main cardiometabolic risk factors is visceral adipose tissue, which promotes the production of pro-inflammatory cytokines and adipokines with cardiodepressive and atherosclerotic properties [28]. The distribution of body fat has different effects on the cardiovascular system. Determining its location and amount—for instance, by bioelectrical impedance analysis or DEXA (dual-energy X-ray absorptiometry)—can facilitate identification of people with a similar BMI but different CVD risk [29,30,31]. Chavda et al. did not link obesity with improved in-hospital survival outcome in patients who were admitted to the ICU after CA [32]. Other researchers have proven that subpar in-hospital mortality and neurological outcomes were the concomitant occurrences among obese CA patients [33]. Obesity is characterized by higher fat mass, which leads to chronic inflammation and a prothrombotic state [34]. Hypoxemia and the decreased functional residual capacity in patients with obesity make them vulnerable to more severe illness, e.g., COVID-19 [35]. Moreover, obesity has been widely recognized as a factor associated with a decrease in the immune response capacity [36]. It is worth mentioning that cardiopulmonary resuscitation of obese patients is more difficult due to issues with performing chest compressions or ventilation [37]. A meta-analysis by Heekyung et al. found that obesity was associated with higher in-hospital mortality. However, underweight was associated with higher in-hospital mortality as well as worse neurological outcomes [38]. The relationship between BMI score and mortality remains unclear, requiring further research.

The results reported in this study did not confirm that a higher risk of malnutrition is associated with mortality in patients who have suffered from CA. However, other researchers have shown that malnutrition was associated not only with a higher in-hospital mortality in the ICU, but also with prolonged hospitalization due to dependence on mechanical ventilation and, consequently, increased medical costs [39,40,41,42]. Critically ill patients, likewise, showed an association between malnutrition and higher mortality [43]. In this study, the status of malnutrition risk was measured using the NRS 2002. This tool is based on BMI, weight loss, severity of the disease, and decreased food intake [23]. A patient admitted to the ICU receives 3 points, which means they are already at risk of malnutrition [23,44]. The use of NRS 2002 in this group of patients requires further research. In our study, hsCRP concentration was positively associated with the odds of death. Coexistent with the chronic state of meta-inflammation, obesity is found among the main factors associated with high CRP [45]. However, our patients were admitted to the ICU for CA. In a study by Dell’anna et al., patients with in-hospital CA and non-shockable rhythms had higher levels of hsCRP compared to patients who suffered from out-of-hospital CA. This could have been due to a hospital stay, which might have increased the risk of infection [46]. CRP is not only commonly used in critically ill patients in order to diagnose new infections or to check the effectiveness of antibiotic therapy. Patients who receive ROSC usually develop ischemia-reperfusion syndrome, which often exacerbates cardiac and brain damage, leading to systemic inflammation—this, together with anoxic brain injury and myocardial dysfunction, is a major component of post-cardiac arrest syndrome (PCAS) [47,48]. Lemiale et al. reported that high numbers of patients who died rapidly after ICU admission severe suffered from PCAS [49]. Though unrelated to nutritional status, a non-defibrillation rhythm was found to be a factor that increased the mortality risk. This is in line with other studies, which confirmed that the chances of survival after CA with a PEA/asystole post rhythm were markedly slim [50,51,52].

### Study Limitations

The study was prone to several limitations. Firstly, the small number of patients in the population sample limited the possibilities to analyze the data with more elaborate methods. However, this was a very specific group of patients who developed ROSC and could be treated in the ICU. Secondly, in some cases the NRS 2002 and BMI scores were not reported in the medical records. Regarding the low proportion (7.5%) of individuals with an NRS ≥5, this could have had an influence on the results. Due to the serious nature of the situation (critically ill patients after CA), complete data concerning drug administration and other information covered by the medical history could prove to be unobtainable. Moreover, either the BIA analysis or waist-to-hip ratio (WHR) and waist circumference measurements were not conducted. It could be assumed that BMI scores might not be a reliable indicator for assessing overweight and obesity. This study was a retrospective analysis. Therefore, obtaining certain data was unfeasible, partially due to the anonymization of patient data, which affected the investigation into long-term survival.

## 5. Conclusions

BMI and NRS 2002 results were not factors which, on their own (unconditionally), altered the odds of mortality in patients admitted to the intensive care unit (ICU) after in-hospital or out-of-hospital cardiac arrest. The risk of in-hospital mortality (expressed as hazard ratio – the risk over the study time period) increased with an increase in BMI but not with NRS 2002. Undoubtedly, the impact of BMI and NRS 2002 results in patients hospitalized in the ICU due to in-hospital and out-of-hospital cardiac arrest requires further investigation.

## Figures and Tables

**Table 1:** Characteristics of the sample population, by BMI value (continuous variables).

Variable	*n*	Total (1st Q, Me, 3rd Q)	*n*	BMI ≥ 30 (1st Q, Me, 3rd Q)	*n*	BMI 25.0–29.9 (1st Q, Me, 3rd Q)	*n*	BMI 18.5–24.9 (1st Q, Me, 3rd Q)	*p*
Age (years)	160	56.00, 67.00, 74.50	37	64.00, 71.00, 74.00	51	57.50, 65.00, 75.50	48	53.00, 66.50, 74.00	0.177
Albumin (g/dL)	136	2.50, 3.00, 3.50	31	2.65, 3.10, 3.60	43	2.75, 3.10, 3.60	43	2.40, 2.80, 3.40	0.221
BMI (kg/m^2^)	129	23.15, 26.23, 30.00	37	30.68, 31.98, 36.73	51	26.12, 27.68, 29.35	48	20.35, 22.67, 23.44	<0.001
HDL (mg/dL)	62	25.00, 34.00, 45.00	17	25.00, 34.00, 46.00	20	29.00, 38.00, 50.25	18	22.00, 30.00, 45.75	0.417
K (mmol/L)	158	3.78, 4.36, 5.09	37	4.13, 4.70, 5.50	51	3.66, 4.27, 4.80	47	3.68, 4.29, 5.46	0.100
LDL (mg/dL)	62	58.00, 83.50, 108.00	17	58.00, 87.00, 134.00	20	68.75, 95.00, 108.00	18	67.50, 83.50, 100.75	0.804
Lymphocytes (%)	73	5.60, 9.40, 16.40	16	6.28, 12.90, 16.25	24	6.52, 10.30, 16.45	23	5.95, 10.40, 15.45	0.903
NRS 2002	161	3.00, 3.00, 4.00	37	3.00, 4.00, 4.00	51	3.00, 3.00, 4.00	48	3.00, 3.00, 4.00	0.152
Na (mmol/L)	158	135.00, 138.00, 141.00	37	135.00, 138.00, 141.00	51	135.00, 139.00, 141.00	47	135.50, 138.00, 142.00	0.680
PCT (ng/mL)	157	0.13, 0.45, 3.61	37	0.13, 0.99, 3.89	51	0.20, 0.51, 3.21	46	0.10, 0.34, 2.24	0.665
TC (mg/dL)	105	111.00, 139.00, 172.00	26	104.00, 136.50, 179.50	38	111.00, 138.50, 163.75	27	112.00, 139.00, 173.00	0.996
TG (mg/dL)	99	91.00, 134.00, 197.00	23	87.00, 156.00, 245.50	36	88.25, 101.50, 193.25	27	103.50, 128.00, 175.50	0.082
TSH (uIU/mL)	99	0.98, 1.67, 3.34	23	1.34, 2.23, 3.20	31	1.09, 1.70, 2.84	30	0.77, 1.72, 3.59	0.655
hsCRP (mg/L)	157	3.19, 13.92, 69.5	37	3.53, 21.90, 128.09	51	3.16, 12.45, 35.70	46	2.70, 14.92, 42.90	0.623

Abbreviations: *n*, number of participants; Q, quartile; Me, median value; *p*, level of significance; BMI, body mass index; HDL, high-density lipoprotein; K, potassium; LDL, low-density lipoprotein; NRS 2002, Nutritional Risk Score; Na, sodium; PCT, procalcitonin; TC, total cholesterol; TG, triglycerides; TSH, thyroid-stimulating hormone; hsCRP, high-sensitivity *C-*reactive protein.

**Table 2:** Characteristics of the sample population and differences in malnutrition risk and obesity (continuous variables).

Variable	*n*	NRS 2002 3–5 (1st Q, Me, 3rd Q)	*n*	NRS 2002 ≥ 5 (1st Q, Me, 3rd Q)	*p*
Age (years)	148	56.75, 67.00, 75.00	12	53.75, 65.50, 72.25	0.846
Albumin (g/dL)	125	2.50, 3.00, 3.50	11	2.15, 2.70, 2.90	0.111
BMI (kg/m^2^)	119	23.44, 26.30, 30.00	10	21.28, 23.26, 26.08	0.040
HDL (mg/dL)	56	24.75, 34.50, 45.25	6	26.75, 30.00, 40.00	0.544
K (mmol/L)	146	3.75, 4.46, 5.11	12	3.79, 3.90, 4.45	0.146
LDL (mg/dL)	56	58.00, 83.50, 108.00	6	74.50, 84.50, 93.75	0.924
Lymphocytes (%)	65	5.60, 9.20, 16.10	8	5.55, 15.20, 16.68	0.751
NRS 2002	149	3.00, 3.00, 4.00	12	5.00, 6.00, 6.00	<0.001
Na (mmol/L)	146	135.25, 138.00, 141.00	12	130.00, 134.50, 141.00	0.149
PCT (ng/mL)	145	0.15, 0.46, 3.62	12	0.07, 0.22, 0.96	0.240
TC (mg/dL)	95	112.00, 140.00, 171.50	10	111.25, 131.50, 172.00	0.943
TG (mg/dL)	89	90.00, 134.00, 197.00	10	107.25, 129.50, 173.25	0.958
TSH (uIU/mL)	93	0.98, 1.66, 3.07	6	1.93, 2.92, 5.47	0.153
hsCRP (mg/L)	145	3.10, 13.92, 80.39	12	3.50, 17.99, 41.79	0.976
**Variable**	***n***	**Non-obese** **(1st Q, Me, 3rd Q)**	***n***	**Obese** **(1st Q, Me, 3rd Q)**	***p***
Age (years)	123	53.00, 65.00, 74.50	37	64.00, 71.00, 74.00	0.039
Albumin (g/dL)	105	2.50, 3.00, 3.50	31	2.65, 3.10, 3.60	0.501
BMI (kg/m^2^)	92	22.49, 24.82, 26.30	37	30.68, 31.98, 36.73	<0.001
HDL (mg/dL)	45	25.00, 34.00, 45.00	17	25.00, 34.00, 46.00	0.925
K (mmol/L)	121	3.71, 4.27, 4.89	37	4.13, 4.70, 5.50	0.021
LDL (mg/dL)	45	62.00, 82.00, 106.00	17	58.00, 87.00, 134.00	0.625
Lymphocytes (%)	57	5.60, 9.20, 16.40	16	6.28, 12.90, 16.25	0.670
NRS 2002	124	3.00, 3.00, 4.00	37	3.00, 4.00, 4.00	0.167
Na (mmol/L)	121	135.00, 138.00, 141.00	37	135.00, 138.00, 141.00	0.800
PCT (ng/mL)	120	0.13, 0.44, 3.01	37	0.13, 0.99, 3.89	0.707
TC (mg/dL)	79	113.00, 140.00, 168.50	26	104.00, 136.50, 179.50	0.917
TG (mg/dL)	76	97.75, 126.50, 194.25	23	87.00, 156.00, 245.50	0.426
TSH (uIU/mL)	76	0.67, 1.65, 3.18	23	1.34, 2.23, 3.20	0.238
hsCRP (mg/L)	120	3.17, 12.54, 41.62	37	3.53, 21.90, 128.09	0.222

Abbreviations: *n*, number of participants; Q, quartile; Me, median value; *p*, level of significance; BMI, body mass index; HDL, high-density lipoprotein; K, potassium; LDL, low-density lipoprotein; NRS 2002, Nutritional Risk Score; Na, sodium; PCT, procalcitonin; TC, total cholesterol; TG, triglycerides; TSH, thyroid-stimulating hormone; hsCRP, high-sensitivity *C-*reactive protein.

**Table 3:** The comparison of assessed parameters (categorical variables) with the ranges of BMI (WHO criteria) values.

Variable	Category	A: BMI 18.5–24.9	B: BMI 25.0–29.9	C: BMI ≥ 30	All	χ2	Global *p*	A: *p*	B: *p*	C: *p*	*p* corr	A: OR	B: OR	C: OR
Sex	Female	17 (0.42)	14 (0.35)	9 (0.22)	40	0.75	0.687	0.405	0.480	0.892	No	1.38	0.76	0.94
	Male	31 (0.35)	37 (0.42)	21 (0.24)	89							0.72	1.32	1.06
	All	48 (0.37)	51 (0.4)	30 (0.23)	129	-	-	-	-	-	-	-	-	-
Cardiac arrest mechanism	Asystole/PEA	30 (0.42)	26 (0.37)	15 (0.21)	71	1.73	0.422	0.190	0.454	0.527	No	1.63	0.76	0.77
	VF/pVT	18 (0.31)	25 (0.43)	15 (0.26)	58							0.62	1.31	1.30
	All	48 (0.37)	51 (0.4)	30 (0.23)	129	-	-	-	-	-	-	-	-	-
Cardiac arrest location	OHCA	23 (0.35)	26 (0.4)	16 (0.25)	65	0.23	0.892	0.666	0.913	0.713	No	0.85	0.96	1.48
	IHCA	25 (0.39)	25 (0.39)	14 (0.22)	64							1.17	1.04	0.68
	All	48 (0.37)	51 (0.4)	30 (0.23)	129	-	-	-	-	-	-	-	-	-
ACS	No ACS	39 (0.38)	41 (0.39)	24 (0.23)	104	0.02	0.989	0.889	0.958	0.922	No	0.85	1.04	1.17
	ACS	9 (0.36)	10 (0.4)	6 (0.24)	25							1.17	0.96	0.86
	All	48 (0.37)	51 (0.4)	30 (0.23)	129	-	-	-	-	-	-	-	-	-
CS	No cerebral stroke	46 (0.39)	48 (0.41)	23 (0.2)	117	9.21	0.010	0.122	0.280	0.003	No	3.24	2.09	0.17
	Cerebral stroke	2 (0.17)	3 (0.25)	7 (0.58)	12	9.21						0.31	0.48	5.72
	All	48 (0.37)	51 (0.4)	30 (0.23)	129	-	-	-	-	-	-	-	-	-
CKD	No CKD	45 (0.38)	49 (0.42)	24 (0.2)	118	6.77	0.034	0.476	0.195	0.031	Yes	1.64	3.20	0.21
	CKD	3 (0.27)	2 (0.18)	6 (0.55)	11	6.77						0.61	0.31	4.70
	All	48 (0.37)	51 (0.4)	30 (0.23)	129	-	-	-	-	-	-	-	-	-
HF	No heart failure	45 (0.4)	45 (0.4)	23 (0.2)	113	4.99	0.083	0.103	0.859	0.038	Yes	2.87	1.10	0.33
	Heart failure	3 (0.19)	6 (0.38)	7 (0.44)	16	4.99						0.35	0.91	3.04
	All	48 (0.37)	51 (0.4)	30 (0.23)	129	-	-	-	-	-	-	-	-	-
DM	No diabetes	43 (0.43)	43 (0.43)	14 (0.14)	100	21.75	<0.001	0.017	0.135	<0.001	Yes	3.62	1.98	0.13
	Diabetes	5 (0.17)	8 (0.28)	16 (0.55)	29	21.75						0.28	0.50	7.56
	All	48 (0.37)	51 (0.4)	30 (0.23)	129	-	-	-	-	-	-	-	-	-
HT	No hypertension	37 (0.46)	29 (0.36)	14 (0.18)	80	8.20	0.017	0.020	0.330	0.072	Yes	2.97	0.70	0.44
	Hypertension	11 (0.22)	22 (0.45)	16 (0.33)	49	8.20						0.34	1.43	2.29
	All	48 (0.37)	51 (0.4)	30 (0.23)	129	-	-	-	-	-	-	-	-	-

The counts of individual strata are shown as observed count (% from table rows). Columns and rows labeled “All” refer to sums of counts from particular columns and labels of the contingency tables. *P*-values associated with 2 × 3 contingency tables created after stratification by BMI are shown in the “global *p*” column. Other *p*-values refer to 2 × 2 contingency tables created from the 2 × 3 tables (e.g., “A: *p*” refers to the A vs. (B + C) comparison). Information on whether FDR correction was used is given in the “*p* corr” column. Abbreviations: OR, odds ratio; ACS, acute coronary syndrome; HF, heart failure; DM, diabetes mellitus; CKD, chronic kidney disease; CS, cerebral stroke; HT, hypertension; OHCA, out-of-hospital cardiac arrest; IHCA, in-hospital cardiac arrest; PEA, pulseless electrical activity; VF, ventricular fibrillation (VF); pVT, pulseless ventricular tachycardia.

**Table 4:** Differences between non-survivors and survivors (continuous variables).

Variable	*n*	Non-Survivors (1st Q, Me, 3rd Q)	*n*	Survivors (1st Q, Me, 3rd Q)	*p*
Age (years)	91	58.50, 67.00, 76.00	69	54.00, 66.00, 73.00	0.259
Albumin (g/dL)	73	2.50, 2.80, 3.30	63	2.75, 3.20, 3.70	0.017
BMI (kg/m^2^)	72	23.15, 26.99, 30.00	57	23.44, 26.23, 30.00	0.632
HDL (mg/dL)	20	22.00, 31.50, 46.00	42	26.50, 35.00, 44.75	0.465
K (mmol/L)	89	3.79, 4.60, 5.20	69	3.78, 4.20, 4.85	0.319
LDL (mg/dL)	20	57.00, 71.00, 112.25	42	70.75, 87.00, 107.50	0.281
Lymphocytes (%)	30	4.32, 8.45, 16.32	43	6.20, 12.00, 16.50	0.326
NRS 2002	92	3.00, 3.00, 4.00	69	3.00, 3.00, 4.00	0.945
Na (mmol/L)	89	135.00, 139.00, 143.00	69	135.00, 138.00, 140.00	0.308
PCT (ng/mL)	88	0.19, 1.04, 4.47	69	0.10, 0.26, 1.52	0.006
TC (mg/dL)	47	97.00, 125.00, 160.50	58	126.75, 153.50, 172.75	0.015
TG (mg/dL)	41	81.00, 134.00, 215.00	58	101.25, 137.50, 193.75	0.430
TSH (uIU/mL)	45	1.14, 2.12, 3.35	54	0.98, 1.62, 3.06	0.391
hsCRP (mg/L)	88	3.48, 18.91, 99.22	69	2.68, 11.09, 51.12	0.161

Abbreviations: *n*, number of participants; Q, quartile; Me, median value; *p*, level of significance; BMI, body mass index; HDL, high-density lipoprotein; K, potassium; LDL, low-density lipoprotein; NRS 2002, Nutritional Risk Score; Na, sodium; PCT, procalcitonin; TC, total cholesterol; TG, triglycerides; TSH, thyroid-stimulating hormone; hsCRP, high-sensitivity *C-*reactive protein.

**Table 5:** Differences between non-survivors and survivors (categorical variables).

Variable	Category	Survivors	Non-Survivors	All	χ2	*p*	OR
Sex	Female	21 (0.4)	32 (0.6)	53	0.34	0.561	1.22
	Male	48 (0.44)	60 (0.56)	108			0.82
	All	69 (0.43)	92 (0.57)	161	-	-	-
Obesity	Non-obese	53 (0.43)	71 (0.57)	124	0.00	0.957	1.02
	Obese	16 (0.43)	21 (0.57)	37			0.98
	All	69 (0.43)	92 (0.57)	161	-	-	-
Cardiac arrest mechanism	Asystole/PEA	29 (0.32)	61 (0.68)	90	9.43	0.002	2.71
	VF/pVT	40 (0.56)	31 (0.44)	71			0.37
	All	69 (0.43)	92 (0.57)	161	-	-	-
Cardiac arrest location	OHCA	34 (0.45)	41 (0.55)	75	0.35	0.553	0.83
	IHCA	35 (0.41)	51 (0.59)	86			1.21
	All	69 (0.43)	92 (0.57)	161	-	-	-
ACS	No ACS	57 (0.43)	77 (0.57)	134	0.03	0.855	1.08
	ACS	12 (0.44)	15 (0.56)	27			0.93
	All	69 (0.43)	92 (0.57)	161	-	-	-
CS	No cerebral stroke	64 (0.44)	83 (0.56)	147	0.32	0.572	0.72
	Cerebral stroke	5 (0.36)	9 (0.64)	14			1.39
	All	69 (0.43)	92 (0.57)	161	-	-	-
CKD	No CKD	61 (0.42)	84 (0.58)	145	0.37	0.543	1.38
	CKD	8 (0.5)	8 (0.5)	16			0.73
	All	69 (0.43)	92 (0.57)	161	-	-	-
HF	No heart failure	63 (0.45)	77 (0.55)	140	2.01	0.156	0.49
	Heart failure	6 (0.29)	15 (0.71)	21			2.05
	All	69 (0.43)	92 (0.57)	161	-	-	-
DM	No diabetes	50 (0.41)	72 (0.59)	122	0.72	0.396	1.37
	Diabetes	19 (0.49)	20 (0.51)	39			0.73
	All	69 (0.43)	92 (0.57)	161	-	-	-
HT	No hypertension	41 (0.4)	61 (0.6)	102	0.80	0.370	1.34
	Hypertension	28 (0.47)	31 (0.53)	59			0.74
	All	69 (0.43)	92 (0.57)	161	-	-	-

The counts of individual strata are shown as observed count (% from table rows). Columns and rows labeled “All” refer to sums of counts from particular columns and labels of the contingency tables. Abbreviations: OR, odds ratio; ACS, acute coronary syndrome; HF, heart failure; DM, diabetes mellitus; CKD, chronic kidney disease; CS, cerebral stroke; HT, hypertension; OHCA, out-of-hospital cardiac arrest; IHCA, in-hospital cardiac arrest; PEA, pulseless electrical activity; VF, ventricular fibrillation (VF); pVT, pulseless ventricular tachycardia.

**Table 6:** Association between selected parameters and odds of death (multivariate logistic regression—all analyzed models).

MODEL 1 (Custom)										
Hosmer–Lemeshow *p*		β = 0 hypothesis *p*			AIC	BIC	Pseudo-R^2^		AUC (learning)	AUC (testing)
0.8471		0.6647			182.26	190.83	0.0085		0.538 ± 0.0511	0.413 ± 0.0507
Effect/interaction	Analyzed cat.	β_i_	β_i_ SE	Wald χ^2^	χ2 −95% CI	χ2 95% CI	*p*	OR	OR −95% CI	OR 95% CI
β_0_ intercept	-	0.538	0.805	0.446	−1.039	2.115	0.504	1.712	0.354	8.288
NRS 2002	-	−0.086	0.222	0.149	−0.522	0.350	0.700	0.918	0.594	1.419
BMI (kg/m^2^)	-	0.022	0.028	0.623	−0.033	0.078	0.430	1.023	0.968	1.081
MODEL 2 (custom)										
Hosmer–Lemeshow *p*		β = 0 hypothesis *p*			AIC	BIC	Pseudo-R^2^		AUC (learning)	AUC (testing)
0.3060		0.6818			184.79	199.09	0.0236		0.579 ± 0.0509	0.379 ± 0.0493
Effect/interaction	Analyzed cat.	β_i_	β_i_ SE	Wald χ^2^	χ2 −95% CI	χ2 95% CI	*p*	OR	OR −95% CI	OR 95% CI
β_0_ intercept	-	−0.323	1.079	0.090	−2.437	1.791	0.764	0.724	0.087	5.995
Sex	Male	0.117	0.199	0.347	−0.273	0.508	0.556	1.125	0.761	1.662
Age (years)	-	0.015	0.013	1.297	−0.011	0.041	0.255	1.015	0.989	1.042
NRS 2002	-	−0.131	0.235	0.312	−0.593	0.330	0.576	0.877	0.553	1.390
BMI (kg/m^2^)	-	0.015	0.029	0.276	−0.041	0.071	0.599	1.015	0.960	1.074
MODEL 3 (stepwise elimination, *p* cutoff = 0.10)										
Hosmer–Lemeshow *p*		β = 0 hypothesis *p*			AIC	BIC	Pseudo-R^2^		AUC (learning)	AUC (testing)
0.209		0.000902			162.64	176.82	0.2042		0.705 ± 0.0492	0.660 ± 0.0520
Effect/interaction	Analyzed cat.	β_i_	β_i_ SE	Wald χ^2^	χ2 −95% CI	χ2 95% CI	*p*	OR	OR −95% CI	OR 95% CI
β_0_ intercept	-	0.594	0.327	3.303	−0.047	1.235	0.069	1.812	0.954	3.439
Cardiac arrest mechanism	VF/pVT	−0.521	0.212	6.026	−0.937	−0.105	0.014	0.594	0.392	0.900
Heart failure	Yes	0.596	0.328	3.298	−0.047	1.240	0.069	1.815	0.954	3.455
hsCRP (mg/L)	-	0.009	0.004	5.495	0.001	0.016	0.019	1.009	1.001	1.016

The “Analyzed cat.” column refers to categories that are compared to reference categories in terms of odds of death. Abbreviations: AIC, Akaike information criterion; BIC, Bayesian information criterion; **β_i_**, regression coefficient; SE, standard error; OR, odds ratio; CI, confidence interval. The “AUC (learning)” and “AUC (testing)” columns show AUC values from tenfold cross-validation; NRS 2002, Nutritional Risk Score; hsCRP, high-sensitivity *C*-reactive protein; BMI, body mass index.

**Table 7:** Multivariate survival analysis (Cox proportional hazards regression).

Variable	Analyzed Cat.	β_i_	β_i_ SE	HR	HR −95% CI	HR 95% CI	*p*
Age (years)	-	0.010	0.012	1.0099	0.9873	1.0331	0.394
NRS 2002	-	−0.200	0.211	0.8189	0.5417	1.2379	0.343
BMI (kg/m^2^)	-	0.062	0.031	1.0637	1.0005	1.1308	0.048
Albumin (g/dL)	-	−0.185	0.195	0.8313	0.5672	1.2183	0.343
K (mmol/L)	-	0.152	0.097	1.1646	0.9629	1.4086	0.116
Na (mmol/L)	-	0.027	0.017	1.0276	0.9929	1.0634	0.120
hsCRP (mg/L)	-	0.006	0.002	1.0060	1.0023	1.0098	0.002
Sex	Female	−0.139	0.154	0.7580	0.4146	1.3859	0.368
Obesity	Non-obese	0.382	0.259	2.1480	0.7772	5.9367	0.140
Cardiac arrest mechanism	Asystole/PEA	0.154	0.168	1.3598	0.7041	2.6264	0.360
Cardiac arrest location	OHCA	−0.005	0.176	0.9909	0.4976	1.9735	0.979
ACS	No ACS	0.131	0.186	1.2997	0.6258	2.6992	0.482
CS	No CS	−0.004	0.262	0.9915	0.3555	2.7648	0.987
CKD	No CKD	0.202	0.259	1.4985	0.5430	4.1349	0.435
HF	No HF	−0.311	0.202	0.5365	0.2435	1.1821	0.122
DM	No DM	0.618	0.249	3.4394	1.2969	9.1214	0.013
HT	No HT	−0.303	0.209	0.5456	0.2403	1.2389	0.148

The “Analyzed cat.” column features the categories compared to their respective reference categories in terms of the hazard function values. Abbreviations: β_i_, regression coefficient; SE, standard error; HR, hazard ratio; CI, confidence interval; ACS, acute coronary syndrome; HF, heart failure; DM, diabetes mellitus; CKD, chronic kidney disease; CS, cerebral stroke; BMI, body mass index; K, potassium; Na, sodium; hsCRP, high-sensitivity *C-*reactive protein; OHCA, out-of-hospital cardiac arrest; PEA, pulseless electrical activity.

## Data Availability

Not applicable.

## Supplementary Materials

The following supporting information can be downloaded at: https://www.mdpi.com/article/10.3390/nu15020436/s1, Table S1: Univariate logistic regression analyzing the odds of death among patients; Table S2: Process of iterative creation of the multivariate logistic regression model (Table 6: model 3); Table S3: Univariate Cox proportional hazard regression.
