# Supplementary material for: Do Body Mass Index and Nutritional Risk Score 2002 Influence the In-Hospital Mortality of Patients Following Cardiac Arrest?
Source: Nutrients. 2023 Jan 14;15(2):436. doi: 10.3390/nu15020436 (PMC9863085; doi:10.3390/nu15020436)
Supplement: Supplementary file 1 [file nutrients-15-00436-s001.zip › nutrients-2154177-supplementary.pdf]

Table S1. Univariate logistic regression analyzing the odds of death among patients

Variable	Analyzed cat.	β_i	β_i SE	OR	OR -95% CI	OR 95% CI	p
**Sex**	**Male**	-0.198	0.341	0.82	0.42	1.60	0.562
**Age [years]**	**-**	0.013	0.011	1.01	0.99	1.04	0.240
**BMI [kg/m^2]**	**-**	0.023	0.028	1.02	0.97	1.08	0.417
**Obesity**	**Obese**	-0.020	0.378	0.98	0.47	2.06	0.957
**Cardiac arrest mechanism**	**VF/SvT**	-0.998	0.329	**0.37**	0.19	0.70	**0.002**
**Cardiac arrest location**	**IHCA**	0.189	0.319	1.21	0.65	2.26	0.553
**ACS**	**ACS**	-0.078	0.425	0.93	0.40	2.13	0.855
**Cerebral stroke**	**Cerebral stroke**	0.328	0.582	1.39	0.44	4.34	0.573
**CKD**	**CKD**	-0.320	0.528	0.73	0.26	2.04	0.544
**Heart failure**	**Heart failure**	0.716	0.512	2.05	0.75	5.58	0.162
**Diabetes**	**Diabetes**	-0.313	0.369	0.73	0.35	1.51	0.396
**Hypertension**	**Hypertension**	-0.296	0.330	0.74	0.39	1.42	0.370
**TG [mg/dl]**	**-**	0.001	0.001	1.00	1.00	1.00	0.368
**TChol [mg/dl]**	**-**	-0.008	0.004	0.99	0.99	1.00	0.063
**Albumin [g/dl]**	**-**	-0.491	0.248	**0.61**	0.38	1.00	**0.048**
**K [mmol/l]**	**-**	0.035	0.119	1.04	0.82	1.31	0.769
**Na [mmol/l]**	**-**	0.006	0.021	1.01	0.97	1.05	0.764
**hsCRP [mg/l]**	**-**	0.004	0.002	1.00	1.00	1.01	0.092
**Procalcitonine [ng/ml]**	**-**	0.003	0.006	1.00	0.99	1.01	0.609
**TSH [uIU/ml]**	**-**	0.125	0.091	1.13	0.95	1.36	0.170
**NRS2002**	**-**	-0.118	0.203	0.89	0.60	1.32	0.561

The 'Analyzed cat.' column features the categories, which are compared to their respective reference categories in terms of the odds of death. βi - regression coefficient; SE - standard error; OR – odds ratio; CI - confidence interval. Abbreviations: ACS, Acute Coronary Syndrome; HF, heart failure; DM, diabetes mellitus; CKD, chronic kidney disease; CS, cerebral stroke; OHCA, out of hospital cardiac arrest; IHCA, in hospital cardiac arrest; PEA, Pulseless Electrical Activity; VF, ventricular fibrillation (VF); pVT, pulseless ventricular tachycardia; BMI, body mass index; HDL, high-density lipoprotein; K, potassium; LDL, low-density lipoprotein; NRS 2002, Nutritional Risk score; Na, sodium; PCT, procalcitonin; TC, total cholesterol; TG, triglycerides; TSH, thyroid-stimulating hormone; hsCRP, high-sensitivity C-reactive protein

Table S2. Process of iterative creation of the multivariate logistic regression model (Table 6: model 3)

Step	Effect (variable)	df	Wald $\chi 2$	Wald p	Score statistic	Score p	Variable status
Step 1	**Sex**	1	1.70	0.192			Included
	**NRS2002 category**	1	0.57	0.450			Included
	**Obesity**	1	0.28	0.595			Included
	**BMI category**	2	2.46	0.293			Included
	**Cardiac arrest mechanism**	1	3.95	0.047			Included
	**Cardiac arrest location**	1	0.08	0.777			Included
	**ACS**	1	0.70	0.403			Included
	**CS**	1	0.79	0.375			Included
	**CKD**	1	0.84	0.358			Included
	**HF**	1	2.12	0.145			Included
	**DM**	1	2.79	0.095			Included
	**HT**	1	0.07	0.786			Included
	**Age [years]**	1	0.00	0.999			Excluded in this step
	**NRS2002**	1	0.29	0.590			Included
	**BMI [kg/m²]**	1	4.54	0.033			Included
	**Albumin [g/dl]**	1	0.37	0.541			Included
	**K [mmol/l]**	1	0.01	0.909			Included
	**Na [mmol/]**	1	0.51	0.475			Included
	**hsCRP [mg/l]**	1	5.16	0.023			Included
	**Procalcitonine [ng/ml]**	1	2.68	0.102			Included
Step 2	**Sex**	1	1.73	0.189			Included
	**NRS2002 category**	1	0.68	0.410			Included
	**Obesity**	1	0.28	0.595			Included
	**BMI category**	2	2.46	0.293			Included
	**Cardiac arrest mechanism**	1	4.37	0.037			Included
	**Cardiac arrest location**	1	0.08	0.771			Included
	**ACS**	1	0.70	0.402			Included
	**CS**	1	0.79	0.374			Included
	**CKD**	1	0.86	0.353			Included
	**HF**	1	2.22	0.136			Included
	**DM**	1	2.79	0.095			Included
	**HT**	1	0.07	0.786			Included
	**Procalcitonine [ng/ml]**	1	2.75	0.097			Included
	**NRS2002**	1	0.35	0.552			Included
	**BMI [kg/m²]**	1	4.58	0.032			Included
	**Albumin [g/dl]**	1	0.37	0.540			Included
	**K [mmol/l]**	1	0.01	0.909			Excluded in this step
	**Na [mmol/]**	1	0.52	0.472			Included
	**hsCRP [mg/l]**	1	5.17	0.023			Included
	**Age [years]**	1			0.00	0.999	Excluded in previous steps
Step 3	**Sex**	1	1.72	0.190			Included
	**NRS2002 category**	1	0.67	0.414			Included
	**Obesity**	1	0.40	0.529			Included
	**BMI category**	2	2.46	0.292			Included
	**Cardiac arrest mechanism**	1	4.42	0.036			Included
	**Cardiac arrest location**	1	0.08	0.778			Included
	**ACS**	1	0.69	0.406			Included

Step	Effect (variable)	df	Wald χ2	Wald p	Score statistic	Score p	Variable status
	**CS**	1	0.79	0.375			Included
	**CKD**	1	0.98	0.322			Included
	**HF**	1	2.31	0.128			Included
	**DM**	1	2.85	0.092			Included
	**HT**	1	0.07	0.789			Excluded in this step
	**Procalcitonine [ng/ml]**	1	2.76	0.097			Included
	**NRS2002**	1	0.35	0.555			Included
	**BMI [kg/m²]**	1	5.07	0.024			Included
	**Albumin [g/dl]**	1	0.36	0.547			Included
	**hsCRP [mg/l]**	1	5.56	0.018			Included
	**Na [mmol/]**	1	0.55	0.457			Included
	**K [mmol/l]**	1			0.01	0.909	Excluded in previous steps
	**Age [years]**	1			<0.01	0.999	Excluded in previous steps
**Step 4**	**Sex**	1	1.65	0.199			Included
	**NRS2002 category**	1	0.67	0.412			Included
	**Obesity**	1	0.36	0.546			Included
	**BMI category**	2	2.40	0.301			Included
	**Cardiac arrest mechanism**	1	4.41	0.036			Included
	**Cardiac arrest location**	1	0.14	0.710			Excluded in this step
	**ACS**	1	0.71	0.401			Included
	**CS**	1	0.80	0.372			Included
	**CKD**	1	0.92	0.338			Included
	**HF**	1	2.76	0.097			Included
	**DM**	1	2.99	0.084			Included
	**Na [mmol/]**	1	0.55	0.458			Included
	**Procalcitonine [ng/ml]**	1	2.81	0.094			Included
	**NRS2002**	1	0.36	0.548			Included
	**BMI [kg/m²]**	1	5.05	0.025			Included
	**Albumin [g/dl]**	1	0.32	0.573			Included
	**hsCRP [mg/l]**	1	5.50	0.019			Included
	**HT**	1			0.07	0.789	Excluded in previous steps
	**K [mmol/l]**	1			0.01	0.915	Excluded in previous steps
	**Age [years]**	1			<0.01	0.991	Excluded in previous steps
**Step 5**	**Sex**	1	1.58	0.208			Included
	**NRS2002 category**	1	0.69	0.408			Included
	**Obesity**	1	0.38	0.535			Excluded in this step
	**BMI category**	2	2.33	0.312			Included
	**Cardiac arrest mechanism**	1	4.57	0.032			Included
	**hsCRP [mg/l]**	1	5.68	0.017			Included
	**ACS**	1	0.77	0.379			Included
	**CS**	1	0.89	0.345			Included
	**CKD**	1	0.82	0.366			Included
	**HF**	1	2.88	0.090			Included
	**DM**	1	3.07	0.080			Included
	**Na [mmol/]**	1	0.57	0.449			Included
	**Procalcitonine [ng/ml]**	1	2.73	0.098			Included
	**NRS2002**	1	0.43	0.511			Included
	**BMI [kg/m²]**	1	5.01	0.025			Included

Step	Effect (variable)	df	Wald χ2	Wald p	Score statistic	Score p	Variable status
	**Albumin [g/dl]**	1	0.47	0.492			Included
	**Cardiac arrest location**	1			0.14	0.709	Excluded in previous steps
	**HT**	1			0.13	0.718	Excluded in previous steps
	**K [mmol/l]**	1			<0.01	0.944	Excluded in previous steps
	**Age [years]**	1			0.01	0.915	Excluded in previous steps
**Step 6**	**Sex**	1	1.88	0.171			Included
	**NRS2002 category**	1	0.61	0.434			Included
	**Albumin [g/dl]**	1	0.64	0.425			Included
	**BMI category**	2	3.69	0.158			Included
	**Cardiac arrest mechanism**	1	4.33	0.038			Included
	**hsCRP [mg/l]**	1	5.43	0.020			Included
	**ACS**	1	0.91	0.341			Included
	**CS**	1	0.90	0.343			Included
	**CKD**	1	0.68	0.410			Included
	**HF**	1	2.61	0.106			Included
	**DM**	1	3.03	0.082			Included
	**Na [mmol/]**	1	0.67	0.414			Included
	**Procalcitonine [ng/ml]**	1	2.90	0.089			Included
	**NRS2002**	1	0.39	0.534			Excluded in this step
	**BMI [kg/m^2]**	1	4.79	0.029			Included
	**Obesity**	1			0.39	0.532	Excluded in previous steps
	**Cardiac arrest location**	1			0.16	0.691	Excluded in previous steps
	**HT**	1			0.09	0.763	Excluded in previous steps
	**K [mmol/l]**	1			0.10	0.754	Excluded in previous steps
	**Age [years]**	1			0.02	0.883	Excluded in previous steps
**Step 7**	**Sex**	1	1.57	0.210			Included
	**NRS2002 category**	1	0.23	0.629			Excluded in this step
	**Albumin [g/dl]**	1	0.61	0.436			Included
	**BMI category**	2	3.94	0.139			Included
	**Cardiac arrest mechanism**	1	4.52	0.033			Included
	**hsCRP [mg/l]**	1	5.57	0.018			Included
	**ACS**	1	0.83	0.363			Included
	**CS**	1	1.07	0.301			Included
	**CKD**	1	0.51	0.475			Included
	**HF**	1	2.82	0.093			Included
	**DM**	1	3.21	0.073			Included
	**Na [mmol/]**	1	0.95	0.329			Included
	**Procalcitonine [ng/ml]**	1	2.66	0.103			Included
	**BMI [kg/m^2]**	1	5.46	0.019			Included
	**NRS2002**	1			0.39	0.534	Excluded in previous steps
	**Obesity**	1			0.34	0.560	Excluded in previous steps
	**Cardiac arrest location**	1			0.23	0.634	Excluded in previous steps
	**HT**	1			0.13	0.722	Excluded in previous steps
	**K [mmol/l]**	1			0.06	0.799	Excluded in previous steps
	**Age [years]**	1			0.17	0.684	Excluded in previous steps
**Step 8**	**Sex**	1	1.57	0.211			Included
	**BMI [kg/m2]**	1	5.69	0.017			Included
	**Albumin [g/dl]**	1	0.56	0.455			Included

Step	Effect (variable)	df	Wald χ2	Wald p	Score statistic	Score p	Variable status
	**BMI category**	2	3.92	0.141			Included
	**Cardiac arrest mechanism**	1	4.88	0.027			Included
	**hsCRP [mg/l]**	1	5.56	0.018			Included
	**ACS**	1	0.96	0.326			Included
	**CS**	1	1.16	0.282			Included
	**CKD**	1	0.47	0.491			Excluded in this step
	**HF**	1	2.84	0.092			Included
	**DM**	1	3.35	0.067			Included
	**Na [mmol/]**	1	1.03	0.310			Included
	**Procalcitonine [ng/ml]**	1	2.69	0.101			Included
	**NRS2002 category**	1			0.23	0.628	Excluded in previous steps
	**NRS2002**	1			0.01	0.934	Excluded in previous steps
	**Obesity**	1			0.31	0.578	Excluded in previous steps
	**Cardiac arrest location**	1			0.14	0.706	Excluded in previous steps
	**HT**	1			0.10	0.752	Excluded in previous steps
	**K [mmol/l]**	1			0.03	0.860	Excluded in previous steps
	**Age [years]**	1			0.17	0.678	Excluded in previous steps
**Step 9**	**Sex**	1	1.61	0.204			Included
	**BMI [kg/m2]**	1	5.29	0.021			Included
	**Albumin [g/dl]**	1	0.64	0.423			Excluded in this step
	**BMI category**	2	3.67	0.160			Included
	**Cardiac arrest mechanism**	1	4.79	0.029			Included
	**hsCRP [mg/l]**	1	5.29	0.021			Included
	**ACS**	1	0.92	0.338			Included
	**CS**	1	1.01	0.315			Included
	**Procalcitonine [ng/ml]**	1	2.67	0.102			Included
	**HF**	1	2.92	0.087			Included
	**DM**	1	3.25	0.071			Included
	**Na [mmol/]**	1	1.00	0.317			Included
	**CKD**	1			0.48	0.489	Excluded in previous steps
	**NRS2002 category**	1			0.20	0.655	Excluded in previous steps
	**NRS2002**	1			0.02	0.899	Excluded in previous steps
	**Obesity**	1			0.20	0.651	Excluded in previous steps
	**Cardiac arrest location**	1			0.04	0.839	Excluded in previous steps
	**HT**	1			0.02	0.902	Excluded in previous steps
	**K [mmol/l]**	1			0.12	0.734	Excluded in previous steps
	**Age [years]**	1			0.16	0.685	Excluded in previous steps
**Step 10**	**Sex**	1	1.54	0.215			Included
	**BMI [kg/m^2]**	1	4.96	0.026			Included
	**Na [mmol/]**	1	0.79	0.375			Excluded in this step
	**BMI category**	2	3.50	0.174			Included
	**Cardiac arrest mechanism**	1	5.27	0.022			Included
	**hsCRP [mg/l]**	1	6.64	0.010			Included
	**ACS**	1	1.00	0.318			Included
	**CS**	1	1.08	0.298			Included
	**Procalcitonine [ng/ml]**	1	2.74	0.098			Included
	**HF**	1	3.42	0.064			Included
	**DM**	1	3.24	0.072			Included

Step	Effect (variable)	df	Wald $\chi 2$	Wald p	Score statistic	Score p	Variable status
	**Albumin [g/dl]**	1			0.65	0.421	Excluded in previous steps
	**CKD**	1			0.57	0.452	Excluded in previous steps
	**NRS2002 category**	1			0.15	0.699	Excluded in previous steps
	**NRS2002**	1			0.01	0.925	Excluded in previous steps
	**Obesity**	1			0.33	0.565	Excluded in previous steps
	**Cardiac arrest location**	1			0.16	0.689	Excluded in previous steps
	**HT**	1			0.00	0.981	Excluded in previous steps
	**K [mmol/l]**	1			0.04	0.833	Excluded in previous steps
	**Age [years]**	1			0.17	0.676	Excluded in previous steps
**Step 11**	**Sex**	1	1.70	0.192			Included
	**BMI [kg/m²]**	1	4.75	0.029			Included
	**DM**	1	3.09	0.079			Included
	**BMI category**	2	3.18	0.204			Included
	**Cardiac arrest mechanism**	1	5.28	0.022			Included
	**hsCRP [mg/l]**	1	6.56	0.010			Included
	**ACS**	1	0.99	0.319			Included
	**CS**	1	0.94	0.332			Excluded in this step
	**Procalcitonine [ng/ml]**	1	2.76	0.097			Included
	**HF**	1	3.12	0.077			Included
	**Na [mmol/]**	1			0.82	0.366	Excluded in previous steps
	**Albumin [g/dl]**	1			0.46	0.499	Excluded in previous steps
	**CKD**	1			0.52	0.470	Excluded in previous steps
	**NRS2002 category**	1			0.21	0.645	Excluded in previous steps
	**NRS2002**	1			<0.01	0.945	Excluded in previous steps
	**Obesity**	1			0.40	0.529	Excluded in previous steps
	**Cardiac arrest location**	1			0.15	0.702	Excluded in previous steps
	**HT**	1			<0.01	0.968	Excluded in previous steps
	**K [mmol/l]**	1			0.14	0.706	Excluded in previous steps
	**Age [years]**	1			0.34	0.557	Excluded in previous steps
**Step 12**	**Sex**	1	2.55	0.110			Included
	**BMI [kg/m²]**	1	4.41	0.036			Included
	**DM**	1	2.38	0.123			Included
	**BMI category**	2	2.72	0.256			Included
	**Cardiac arrest mechanism**	1	5.54	0.019			Included
	**hsCRP [mg/l]**	1	6.58	0.010			Included
	**ACS**	1	1.16	0.282			Excluded in this step
	**HF**	1	3.03	0.082			Included
	**Procalcitonine [ng/ml]**	1	2.49	0.115			Included
	**CS**	1			0.96	0.327	Excluded in previous steps
	**Na [mmol/]**	1			0.64	0.425	Excluded in previous steps
	**Albumin [g/dl]**	1			0.53	0.467	Excluded in previous steps
	**CKD**	1			0.36	0.546	Excluded in previous steps
	**NRS2002 category**	1			0.29	0.593	Excluded in previous steps
	**NRS2002**	1			<0.01	0.958	Excluded in previous steps
	**Obesity**	1			0.39	0.531	Excluded in previous steps
	**Cardiac arrest location**	1			0.31	0.578	Excluded in previous steps
	**HT**	1			0.01	0.907	Excluded in previous steps
	**K [mmol/l]**	1			0.07	0.788	Excluded in previous steps

Step	Effect (variable)	df	Wald χ2	Wald p	Score statistic	Score p	Variable status
	**Age [years]**	1			0.45	0.505	Excluded in previous steps
**Step 13**	**Sex**	1	2.30	0.129			Included
	**BMI [kg/m²]**	1	4.39	0.036			Included
	**DM**	1	2.45	0.118			Included
	**BMI category**	2	2.70	0.259			Excluded in this step
	**Cardiac arrest mechanism**	1	5.24	0.022			Included
	**hsCRP [mg/l]**	1	6.81	0.009			Included
	**Procalcitonine [ng/ml]**	1	2.59	0.108			Included
	**HF**	1	2.54	0.111			Included
	**ACS**	1			1.17	0.278	Excluded in previous steps
	**CS**	1			1.13	0.288	Excluded in previous steps
	**Na [mmol/]**	1			0.64	0.425	Excluded in previous steps
	**Albumin [g/dl]**	1			0.63	0.429	Excluded in previous steps
	**CKD**	1			0.29	0.588	Excluded in previous steps
	**NRS2002 category**	1			0.46	0.499	Excluded in previous steps
	**NRS2002**	1			0.04	0.836	Excluded in previous steps
	**Obesity**	1			0.59	0.444	Excluded in previous steps
	**Cardiac arrest location**	1			0.41	0.520	Excluded in previous steps
	**HT**	1			0.02	0.878	Excluded in previous steps
	**K [mmol/l]**	1			0.03	0.864	Excluded in previous steps
	**Age [years]**	1			0.55	0.456	Excluded in previous steps
**Step 14**	**Sex**	1	2.11	0.146			Included
	**BMI [kg/m²]**	1	2.05	0.152			Excluded in this step
	**DM**	1	2.54	0.111			Included
	**HF**	1	2.48	0.116			Included
	**Cardiac arrest mechanism**	1	5.33	0.021			Included
	**hsCRP [mg/l]**	1	6.13	0.013			Included
	**Procalcitonine [ng/ml]**	1	2.26	0.133			Included
	**BMI category**	2			2.79	0.248	Excluded in previous steps
	**ACS**	1			1.12	0.290	Excluded in previous steps
	**CS**	1			0.51	0.477	Excluded in previous steps
	**Na [mmol/]**	1			0.32	0.572	Excluded in previous steps
	**Albumin [g/dl]**	1			0.46	0.498	Excluded in previous steps
	**CKD**	1			0.11	0.745	Excluded in previous steps
	**NRS2002 category**	1			0.42	0.515	Excluded in previous steps
	**NRS2002**	1			0.01	0.926	Excluded in previous steps
	**Obesity**	1			1.94	0.164	Excluded in previous steps
	**Cardiac arrest location**	1			0.29	0.590	Excluded in previous steps
	**HT**	1			0.01	0.915	Excluded in previous steps
	**K [mmol/l]**	1			0.14	0.711	Excluded in previous steps
	**Age [years]**	1			0.47	0.492	Excluded in previous steps
**Step 15**	**Sex**	1	2.29	0.130			Included
	**Procalcitonine [ng/ml]**	1	2.75	0.097			Included
	**DM**	1	1.10	0.294			Excluded in this step
	**HF**	1	3.11	0.078			Included
	**Cardiac arrest mechanism**	1	4.76	0.029			Included
	**hsCRP [mg/l]**	1	5.81	0.016			Included
	**BMI [kg/m²]**	1			2.11	0.146	Excluded in previous steps

Step	Effect (variable)	df	Wald χ2	Wald p	Score statistic	Score p	Variable status
	**BMI category**	2			0.02	0.991	Excluded in previous steps
	**ACS**	1			1.06	0.304	Excluded in previous steps
	**CS**	1			0.57	0.450	Excluded in previous steps
	**Na [mmol/]**	1			0.47	0.495	Excluded in previous steps
	**Albumin [g/dl]**	1			0.17	0.678	Excluded in previous steps
	**CKD**	1			<0.01	0.970	Excluded in previous steps
	**NRS2002 category**	1			0.86	0.355	Excluded in previous steps
	**NRS2002**	1			<0.01	0.907	Excluded in previous steps
	**Obesity**	1			0.00	0.945	Excluded in previous steps
	**Cardiac arrest location**	1			0.24	0.624	Excluded in previous steps
	**HT**	1			0.07	0.787	Excluded in previous steps
	**K [mmol/l]**	1			0.20	0.656	Excluded in previous steps
	**Age [years]**	1			0.96	0.327	Excluded in previous steps
**Step 16**	**Sex**	1	2.38	0.123			Excluded in this step
	**Procalcitonine [ng/ml]**	1	2.58	0.108			Included
	**hsCRP [mg/l]**	1	5.44	0.020			Included
	**HF**	1	2.61	0.106			Included
	**Cardiac arrest mechanism**	1	4.90	0.027			Included
	**DM**	1			1.12	0.291	Excluded in previous steps
	**BMI [kg/m²]**	1			0.57	0.450	Excluded in previous steps
	**BMI category**	2			0.18	0.916	Excluded in previous steps
	**ACS**	1			1.19	0.276	Excluded in previous steps
	**CS**	1			0.11	0.741	Excluded in previous steps
	**Na [mmol/]**	1			0.34	0.558	Excluded in previous steps
	**Albumin [g/dl]**	1			0.23	0.633	Excluded in previous steps
	**CKD**	1			0.02	0.879	Excluded in previous steps
	**NRS2002 category**	1			0.77	0.379	Excluded in previous steps
	**NRS2002**	1			0.01	0.905	Excluded in previous steps
	**Obesity**	1			0.27	0.605	Excluded in previous steps
	**Cardiac arrest location**	1			0.25	0.617	Excluded in previous steps
	**HT**	1			0.09	0.759	Excluded in previous steps
	**K [mmol/l]**	1			0.29	0.590	Excluded in previous steps
	**Age [years]**	1			0.57	0.451	Excluded in previous steps
**Step 17**	**Cardiac arrest mechanism**	1	3.58	0.058			Included
	**Procalcitonine [ng/ml]**	1	2.11	0.146			Excluded in this step
	**hsCRP [mg/l]**	1	5.13	0.024			Included
	**HF**	1	2.72	0.099			Included
	**Sex**	1			2.42	0.120	Excluded in previous steps
	**DM**	1			1.21	0.271	Excluded in previous steps
	**BMI [kg/m²]**	1			0.65	0.422	Excluded in previous steps
	**BMI category**	2			0.07	0.966	Excluded in previous steps
	**ACS**	1			0.98	0.323	Excluded in previous steps
	**CS**	1			0.41	0.522	Excluded in previous steps
	**Na [mmol/]**	1			0.42	0.519	Excluded in previous steps
	**Albumin [g/dl]**	1			0.16	0.689	Excluded in previous steps
	**CKD**	1			0.04	0.850	Excluded in previous steps
	**NRS2002 category**	1			0.84	0.360	Excluded in previous steps
	**NRS2002**	1			0.15	0.698	Excluded in previous steps

Step	Effect (variable)	df	Wald χ2	Wald p	Score statistic	Score p	Variable status
	**Obesity**	1			0.27	0.605	Excluded in previous steps
	**Cardiac arrest location**	1			0.12	0.727	Excluded in previous steps
	**HT**	1			0.34	0.561	Excluded in previous steps
	**K [mmol/l]**	1			0.32	0.573	Excluded in previous steps
	**Age [years]**	1			0.13	0.718	Excluded in previous steps
**Step 18**	**Cardiac arrest mechanism**	1	6.03	0.014			Included
	**HF**	1	3.30	0.069			Included
	**hsCRP [mg/l]**	1	5.50	0.019			Included
	**Procalcitonine [ng/ml]**	1			2.59	0.108	Excluded in previous steps
	**Sex**	1			2.55	0.110	Excluded in previous steps
	**DM**	1			1.37	0.242	Excluded in previous steps
	**BMI [kg/m²]**	1			0.90	0.343	Excluded in previous steps
	**BMI category**	2			0.16	0.924	Excluded in previous steps
	**ACS**	1			0.83	0.362	Excluded in previous steps
	**CS**	1			0.25	0.614	Excluded in previous steps
	**Na [mmol/]**	1			0.68	0.408	Excluded in previous steps
	**Albumin [g/dl]**	1			0.23	0.633	Excluded in previous steps
	**CKD**	1			0.08	0.783	Excluded in previous steps
	**NRS2002 category**	1			0.95	0.330	Excluded in previous steps
	**NRS2002**	1			0.43	0.511	Excluded in previous steps
	**Obesity**	1			0.30	0.583	Excluded in previous steps
	**Cardiac arrest location**	1			0.02	0.888	Excluded in previous steps
	**HT**	1			0.57	0.449	Excluded in previous steps
	**K [mmol/l]**	1			0.44	0.509	Excluded in previous steps
	**Age [years]**	1			0.00	0.944	Excluded in previous steps

Abbreviations: ACS, Acute Coronary Syndrome; HF, heart failure; DM, diabetes mellitus; CKD, chronic kidney disease; CS, cerebral stroke; OHCA, out of hospital cardiac arrest; IHCA, in hospital cardiac arrest; PEA, Pulseless Electrical Activity; VF, ventricular fibrillation (VF); pVT, pulseless ventricular tachycardia; BMI, body mass index; HDL, high-density lipoprotein; K, potassium; LDL, low-density lipoprotein; NRS 2002, Nutritional Risk score; Na, sodium; PCT, procalcitonin; TC, total cholesterol; TG, triglycerides; TSH, thyroid-stimulating hormone; hsCRP, high-sensitivity C-reactive protein

Table S3. Univariate Cox proportional hazard regression

Variable	Analyzed cat.	β_i	β_i SE	HR	HR -95% CI	HR 95% CI	p
**TG [mg/dl]**	**-**	0.002	0.001	**1.0023**	1.0004	1.0043	**0.017**
**hsCRP [mg/l]**	**-**	0.003	0.001	**1.0034**	1.0006	1.0063	**0.018**
**Albumin [g/dl]**	**-**	-0.294	0.158	0.7452	0.5471	1.0149	0.062
**Cardiac arrest mechanism**	**Asystole/PEA**	0.186	0.110	1.4495	0.9401	2.2349	0.093
**Diabetes**	**No diabetes**	0.188	0.127	1.4554	0.8857	2.3918	0.139
**Na [mmol/l]**	**-**	0.018	0.014	1.0183	0.9915	1.0458	0.182
**K [mmol/l]**	**-**	0.089	0.071	1.0933	0.9508	1.2572	0.211
**Age [years]**	**-**	0.008	0.007	1.0083	0.9941	1.0226	0.252
**TSH [uIU/ml]**	**-**	0.035	0.030	1.0354	0.9754	1.0991	0.254
**TChol [mg/dl]**	**-**	-0.003	0.003	0.9967	0.9910	1.0025	0.263
**Cardiac arrest location**	**OHCA**	-0.116	0.105	0.7935	0.5249	1.1996	0.273
**Hypertension**	**No hypertension**	0.102	0.110	1.2268	0.7957	1.8915	0.355
**Heart failure**	**No heart failure**	-0.114	0.141	0.7957	0.4572	1.3848	0.419
**NRS2002**	**-**	-0.092	0.136	0.9124	0.6987	1.1915	0.501
**Procalcitonine [ng/ml]**	**-**	0.002	0.003	1.0018	0.9961	1.0076	0.536
**CKD**	**No CKD**	0.114	0.185	1.2555	0.6073	2.5957	0.539
**Obesity**	**Non-obese**	0.061	0.125	1.1297	0.6929	1.8417	0.625
**Sex**	**Female**	0.040	0.110	1.0835	0.7053	1.6645	0.714
**Cerebral stroke**	**No cerebral stroke**	-0.061	0.176	0.8860	0.4451	1.7637	0.730
**ACS**	**No ACS**	0.042	0.142	1.0878	0.6244	1.8949	0.766
**BMI [kg/m^2]**	**-**	0.003	0.016	1.0028	0.9712	1.0353	0.866

The 'Analyzed cat.' column features the categories, which are compared to their respective reference categories in terms of the hazard function values. βi - regression coefficient; SE - standard error; HR - hazard ratio; CI - confidence interval. Abbreviations: Abbreviations: ACS, Acute Coronary Syndrome; HF, heart failure; DM, diabetes mellitus; CKD, chronic kidney disease; CS, cerebral stroke; OHCA, out of hospital cardiac arrest; IHCA, in hospital cardiac arrest; PEA, Pulseless Electrical Activity; VF, ventricular fibrillation (VF); pVT, pulseless ventricular tachycardia; BMI, body mass index; HDL, high-density lipoprotein; K, potassium; LDL, low-density lipoprotein; NRS 2002, Nutritional Risk score; Na, sodium; PCT, procalcitonin; TC, total cholesterol; TG, triglycerides; TSH, thyroid-stimulating hormone; hsCRP, high-sensitivity C-reactive protein
